# On-treatment risk model for predicting treatment response in advanced renal cell carcinoma

**DOI:** 10.1007/s00345-023-04545-2

**Published:** 2023-08-08

**Authors:** Melis Guer, Andreas Janitzky, Martin Schostak

**Affiliations:** Clinic of Urology, Uro-Oncology, Robot-Assisted and Focal Therapy, University Hospital Magdeburg, Magdeburg, Germany

**Keywords:** Advanced renal cell carcinoma, C-Reactive protein, Predictive biomarker, Checkpoint inhibitors, Treatment-related adverse events

## Abstract

**Purpose:**

The field of immunotherapy combinations for advanced renal cell carcinoma (aRCC) has been expanded in recent years. However, the treatment response varies widely among individual patients. It is still a challenge to predict oncological outcome in clinical practice. We assessed the impact of an activated immune system reflected by changes in C-reactive protein (CRP) levels and the early onset of treatment-related adverse events (TRAEs) on the treatment response.

**Methods:**

In this retrospective analysis of 57 aRCC patients, CRP kinetics based on previous descriptions of CRP flare-response, CRP response or CRP non-response, and the TRAEs, which occurred within a month after therapy initiation, were obtained for this study. According to logistic regression analysis of both factors, we stratified the patients into risk groups: the presence of CRP flare-response/response and early onset of TRAE (low-risk group); the presence of a single factor (intermediate-risk group); and without both factors (high-risk group).

**Results:**

Ten patients (17%) experienced primary disease progression. No progressive disease was observed in the low-risk group, while 60% (*n* = 6/10) of the high-risk group showed a primary disease progression. Significantly, an increased risk of disease progression was observed by patients without CRP response and TRAEs (*p* < 0.001).

**Conclusion:**

The present analysis displays the predictive value of the on-treatment risk model based on CRP kinetics and the early onset of TRAEs, which can be easy to implement in clinical practice to optimize the treatment monitoring.

## Introduction

The treatment landscape for advanced renal cell carcinoma (aRCC) has revolutionized in recent years with the introduction of checkpoint blockade antibodies targeting cytotoxic T lymphocyte antigen 4 (CTLA-4), programmed cell death 1 (PD-1) and its ligands such as programmed cell death ligand 1 (PD-L1). Following the approved immunotherapy (IO)/IO combination with the anti-PD-1 antibody Nivolumab plus anti-CTLA-4 antibody Ipilimumab in the first-line therapy, the treatment of IO/Tyrosine kinase inhibitor (TKI) combination for the first-line treatment has gained increasing attention with the improved consistent clinical efficacy [1–3]. Recently, international guidelines have suggested a combination of two of these agents (IO/IO or IO/TKI) as the best strategy to treat aRCC [4, 5]. Although the significant benefits of IO-based combination treatment are proven, the treatment response varies widely among individual patients. In addition, there is still a subset of patients with primary treatment failure. To recognize the antitumor response to immunotherapy might be essential to observe the first immune activity of patients after therapy initiation.

The CRP value is an unspecific measurable marker of systemic inflammation. The association between CRP and renal cell carcinoma has been studied often in the last decade, including intratumoral CRP expression and preoperative or pre-therapeutic CRP levels as a biomarker for survival [6–8]. Importantly, recent studies have investigated CRP kinetics within three months after therapy initiation and described the on-treatment CRP kinetics with three subgroups such as CRP responders, CRP flare-responders, and CRP non-responders [9–11]. The emerging CRP flare kinetic was defined as an early doubling of baseline CRP followed by a drop below baseline by Fukuda et al. (2021), and its predictive value has just recently been validated in a Phase III pivotal clinical trial for non-small cell lung cancer [12]. These studies have shown a significant association between CRP flare-response and treatment efficacy with a favorable oncological outcome.

As a result of the increasing number of aRCC patients in the first-line IO-based combination treatment, there is a wide range of treatment-related adverse events (TRAEs) to manage in clinical practice [13]. According to previous studies, TRAEs may predict better antitumor response following an increased immune activity after the initiation of immunotherapy [14, 15]. In addition, several studies have investigated hypertension as a potential biomarker of TKI efficacy [16].

We hypothesized that the early CRP kinetics, in combination with the early onset of TRAEs, may have higher predictive value for treatment response and oncological outcome. In this regard, we aimed to investigate the predictive impact of the above-mentioned factors on the primary antitumor response to improve clinical monitoring and to achieve more comprehensive oncological care of aRCC patients.

## Methods

We retrospectively reviewed the available medical data from 57 patients treated with the first-line PD-1-based immunotherapy combination (IO/IO or IO/TKI) by aRCC at our institution from July 2015 to June 2023. The combination therapy was administrated according to standard protocols until disease progression, death, or intolerable adverse events. The administration interval could be varied based on the patient`s general condition. According to International Metastatic Renal Cell Carcinoma Database Consortium (IMDC), the prognostic classification was performed for all patients. The ethics committee approved the study protocol (109/22). Considering the retrospective design, subsequent informed consent was waived. No data that would have allowed a reference to the individual patient were used for the analysis.

Data of serum CRP levels were available for all patients: (1) before starting the treatment; (2) at least one time within a month after therapy initiation; (3) two times during further applications within 3 months. Serum CRP levels (in mg/L) were measured in accredited routine laboratories. For this study, patients were divided into three groups based on their CRP kinetics as defined by Fukuda et al. [1]: CRP flare-responders, doubling of baseline CRP within the first month and drop at least once within three months; CRP responders, at least 30% decrease of baseline CRP without prior flare; and the remaining patients as CRP non-responders.

At every administration of immunotherapy for each patient, the occurrence of adverse events was recorded. The time to the first occurrence of AEs obtained from the patient files for this study. For each TRAE, the severity grading was assessed according to Common Terminology Criteria for Adverse Events (CTCAE) version 5.0. According to computations of the mean time to the first occurrence, we defined the early onset of TRAEs within 4 weeks after treatment initiation.

All patients had measurable advanced tumor disease, detected by pre-therapeutic standard imaging exams such as CT scans or MRIs. Response or progression to treatment was assessed according to the RECIST v1.1 criteria. The primary treatment response at the first radiological disease assessment was compared in the subgroups of CRP kinetics and the risk groups of the on-treatment risk model for the primary endpoint of the study. The objective response rate (ORR) refers to the proportion of patients showing partial or complete response to therapy.

**Fig. 1:**
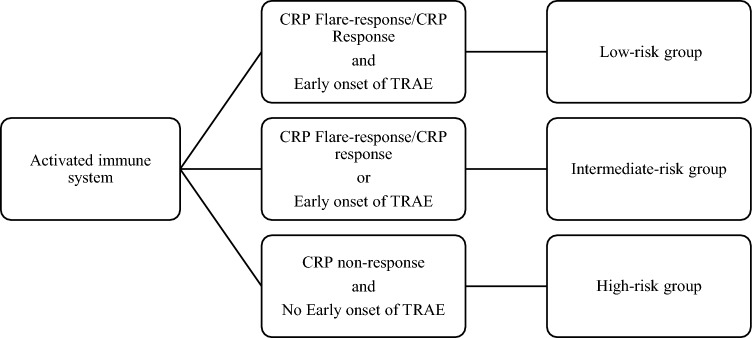
On-treatment risk model with three defined risk groups

The binomial logistic regression was used to analyze the association between CRP-Response, early onset of TRAEs, and the primary treatment response. According to the results by clarification of the statistical significance and R-squared (R^2^) measures, we establish an on-treatment model with three distinct risk groups to predict the primary treatment response (Fig. 1).

The comparison of defined risk groups on primary treatment response was analyzed with Fisher’s exact test. The progression-free survival (PFS) was defined from treatment initiation until death, tumor progression or last observed day was estimated using the Kaplan–Meier method and differences between the groups were assessed using Wilcoxon test. We calculated the Hazard Ratio using a Cox proportional hazards regression and the Odds Ratio using logistic regression analysis. All analyses were conducted using Statistical Package for Social Sciences (SPSS) Version 28 für Windows. Two-sided *P* < 0.05 was considered statistically significant.

## Results

### Characteristics of the study cohort

A total of 57 aRCC patients treated with first-line immunotherapy combination (IO/IO or IO/TKI) between July 2015 and June 2023 were analyzed in this study. The mean age at the treatment initiation was 67 years (44–85). Males comprised 68% of patients. The median duration of treatment until the first radiological disease assessment was 12 weeks (9–28 weeks), while the median follow-up period was 8 months (2–41 months). The 84% of patients (*n* = 48) treated with IO/TKI combinations: 24 patients with Pembrolizumab plus Axitinib (42%); 20 patients with Nivolumab plus Cabozantinib (35%); 2 patients each with Avelumab plus Axitinib (3.5%) and Pembrolizumab plus Lenvatinib (3.5%). *n* = 48 patients were diagnosed with clear cell RCC (84%), and *n* = eight patients with papillary RCC (14%). There was a single patient with sarcomatoid differentiation of clear cell RCC.

The prognostic classification according to IMDC-Score was observed by 13 patients with favorable risk (23%), 28 patients with intermediate risk (49%), and 16 patients with poor risk (28%).

At the initial disease assessment after treatment initiation, ten patients experienced primary disease progression. There was only one patient who has a complete response, while the partial response and stable disease occurred in 29 (50%) and 18 (31%) patients, respectively. Overall, disease progression was observed in 53% (*n* = 30/57) of the patients. The baseline characteristics of the study cohort and their association with primary treatment response are presented in Table 1.

**Table 1:** Baseline characteristics of the study cohort in comparison with the outcome of primary treatment response. *p-values are from Fisher’s exact tests

			Primary treatment response			
Variable		Overall (*n* = 57)	Progressive disease (*n* = 10)	Stable disease (*n* = 18)	Partial/complete response (*n* = 29)	*p*-value*
Age (years)	Median (IQR)	67 (44–85)	64.5 (53–80)	68.5 (55–85)	67 (44–85)	0.081
Sex	Male	39 (68%)	5 (50%)	17 (94%)	17 (58%)	0.006
	Female	18 (32%)	5 (50%)	1 (6%)	12 (42%9	
ECOG PS	0	32 (56%)	5 (50%)	8 (44%)	19 (65%)	0.333
	≥ 1	25 (44%)	5 (50%)	10 (56%)	10 (35%)	
IMDC risk	Favorable	13 (23%)	1 (10%)	2 (11%)	10 (35%)	0.305
	Intermediate	28 (49%)	6 (60%)	10 (56%)	12 (41%)	
	Poor	16 (28%)	3 (30%)	6 (33%)	7 (24%)	
Histology	Clear cell	48 (84%)	8 (80%)	15 (83%)	25 (86%)	0.893
	Non clear cell	9 (16%)	2 (20%)	3 (17%)	4 (14%)	
First-line immunotherapy combinations	IO/IO	9 (16%)	5 (50%)	3 (17%)	1 (3%)	0.004
	IO/TKI	48 (84%)	5 (50%)	15 (83%)	28 (97%)	
CRP kinetics	CRP flare-response	9 (16%)	1 (10%)	1 (5%)	7 (24%)	0.022
	CRP response	22 (38%)	1 (10%)	7 (39%)	14 (48%)	
	CRP Non-response	26 (46%)	8 (80%)	10 (56%)	8 (28%)	
Time to first occurrence of TRAE	≤ 4 weeks	31 (54%)	2 (20%)	15 (83%)	14 (48%)	0.002
	≥ 4 weeks	26 (46%)	8 (80%)	3 (17%)	15 (52%)	
On-treatment risk model	Favorable-risk group	15 (26%)	–	5 (23%)	10 (34%)	< 0.001
	Intermediate-risk group	32 (56%)	4 (40%)	13 (72%)	15 (52%)	
	Poor-risk group	10 (18%)	6 (60%)	–	4 (14%)	

### Early CRP kinetics

**Fig. 2:**
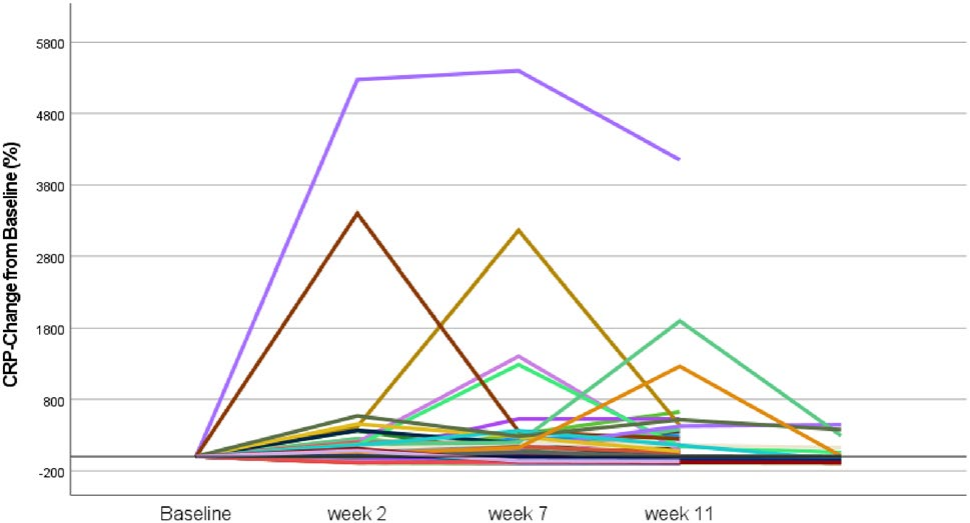
Longitudinal CRP changes from baseline after treatment initiation within 3 months

The percentage changes of CRP levels and the mean time of assessed CRP values from baseline are calculated for each patient. The individual CRP kinetics is depicted in Fig. 2.

**Fig. 3:**
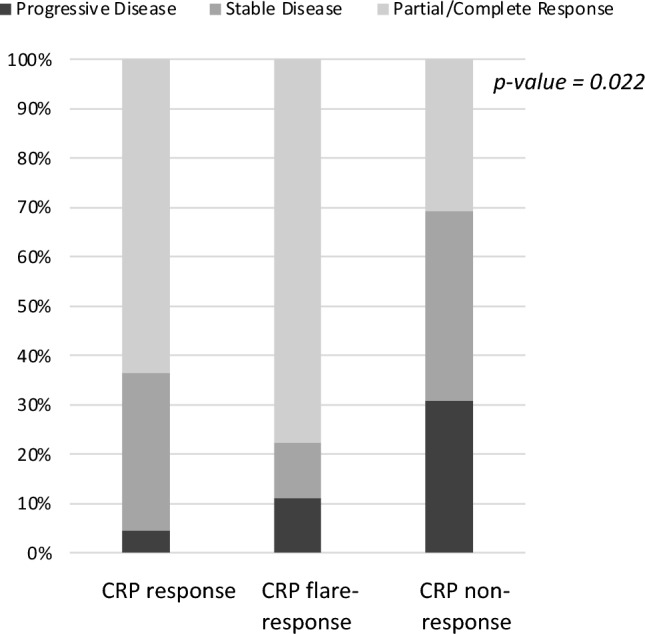
Association between primary treatment response and the subgroups of CRP kinetics

According to the validated description of on-treatment CRP kinetics, CRP flare-response was observed by 9 patients (15.8%), CRP response by 22 patients (38.6%), and CRP non-response by 26 patients (45.6%). The mean time of the assessed first on-treatment CRP value in the overall study population and the subgroup with CRP flare-response was 2 weeks. In comparison to patients with CRP non-response, the patients with CRP flare-response and CRP response were 7.95 times (95% CI [1.32; 47.89]) and 4.24 times (95% CI [1.23; 14.52]) more likely to have an objective response at the first radiological disease assessment, respectively (*p* = 0.020). The primary treatment response differed significantly among the subgroups of CRP kinetics (Figure 3). The objective response (partial and complete response) at the initial disease assessment was predominantly observed in the subgroup of CRP flare-response and CRP response, while 80% (*n* = 8/10) of patients with primary progressive disease was in the subgroup of CRP non-response (*p* = 0.022) (Fig. 3). In the univariate Cox regression, CRP kinetics was not significantly associated with PFS and OS (Table 2).

**Table 2:** Prognostic association of CRP kinetics, early onset of TRAE, and several baseline characteristics for the treatment outcome. Univariable cox regression results and hazard ratios for PFS and OS and logistic regression results and odds ratios for the objective response rate

	ORR		PFS		OS	
Variable	OR	95% CI (*p*)	HR	95% CI (*p*)	HR	95% CI (*p*)
Age > 67	2.59	0.60–11.06(*p* = 0.198)	0.63	0.30–1.33(*p* = 0.232)	0.65	0.17–2.45(*p* = 0.532)
Female gender	1.9	0.44–8.72(*p* = 0.373)	1.12	0.53–2.37(*p* = 0.756)	2.12	0.63–7.07(*p* = 0.219)
ECOG > 1	0.47	0.11–2.02(*p* = 0.315)	1.15	0.55–2.40(*p* = 0.695)	1.15	0.26–4.95(*p* = 0.846)
CRP kinetics						
CRP flare-response	4.87	0.73–32.46(*p* = 0.101)	0.75	0.24–2.34(*p* = 0.623)	0.58	0.13–2.61(*p* = 0.483)
CRP response	10.4	2.00–54.56(*p* = 0.005)	0.94	0.42–2.08(*p* = 0.890)	1.25	0.31–5.03(*p* = 0.751)
CRP non-response	Ref	Ref	Ref	Ref	Ref	Ref
TRAE ≤ 4 weeks	2.27	0.58–8.91(*p* = 0.238)	0.54	0.25–1.12(*p* = 0.101)	0.66	0.20–2.13(*p* = 0.487)

*CI* confidence interval, *CRP* C-reactive protein, *OR* odds ratio, *HR* hazard ratio, *ORR* objective response rate, *PFS* progression-free survival, *OS* overall survival

### Overview of observed TRAEs

All patients experienced at least one TRAE. The mean time to the first occurrence of treatment-related adverse event (TRAE) was 4 weeks (1–28 weeks) after treatment initiation. Among individual patients, the cutaneous TRAEs were experienced mainly at the time of initial occurrence. Specifically, cutaneous, cardiovascular, gastrointestinal, and endocrine TRAEs occurred in 24% (*n* = 14), 16% (*n* = 9), 14% (*n* = 8), and 11% (*n* = 6) of patients, respectively. In most cases, patients experienced moderate adverse events (CTCAE grade 2) at the initial occurrence of TRAEs. There were 21 patients (36%) with grade 1 adverse events and 30 (53%) with grade 2 adverse events, while grade 3 occurred in 20% of patients.

The patients with early onset of TRAEs were more likely to have an objective response (OR 2.27, 95% CI 0.58–8.91, *p* = 0.238) and less likely to have a progression (HR 0.54, 95% CI 0.25–1.12, *p* = 0.101). Of note, there was no significant difference (Table 2).

In 54% (*n* = 31) of the study cohort, TRAEs occurred within 4 weeks after treatment initiation—the overview of observed adverse events within 4 weeks after treatment initiation is listed in Table 3.

**Table 3:** The overview of observed adverse events within 4 weeks after treatment initiation

Early occurrence of TRAEs	*n* (%)	CTCAE grade
Overall	30 (52, 6)	
General disorders	8 (14,0)	1–3
Cutaneous	8 (14,0)	1–2
Cardiovascular	5 (8,8)	1–2
Gastroinstestinal	4 (7,0)	1–2
Pulmonal	1 (1,8)	1
Neurological	1 (1,8)	1
Endocrine	1 (1,8)	1
Urinary	1 (1,8)	1

*TRAEs* treatment-related adverse events, *CTCAE* Common Terminology Criteria for Adverse Events

### Construction of the on-treatment model to predict treatment response

We selected CRP kinetics (CRP flare-response/CRP response/CRP non-response) and the early onset of TRAEs as variables to construct a predictive model for primary treatment response in the first-line treatment of aRCC patients. We performed the forward selection and backward elimination methods to examine the statistical significance of variable selection to include in a regression model. CRP kinetics contributed significantly to predicting treatment response, especially CRP flare-response OR = 7.950 (95% CI [1.320, 47.894]) (*p* = 0.024). The logistic regression model was statistically significant, *χ*^2^ (5) = 8.378, *p* = 0.015. The Nagelkerke *R*^*2*^ value of 0.182 indicated that the model based on CRP kinetics and TRAEs significantly predicts the primary treatment response.

**Fig. 4:**
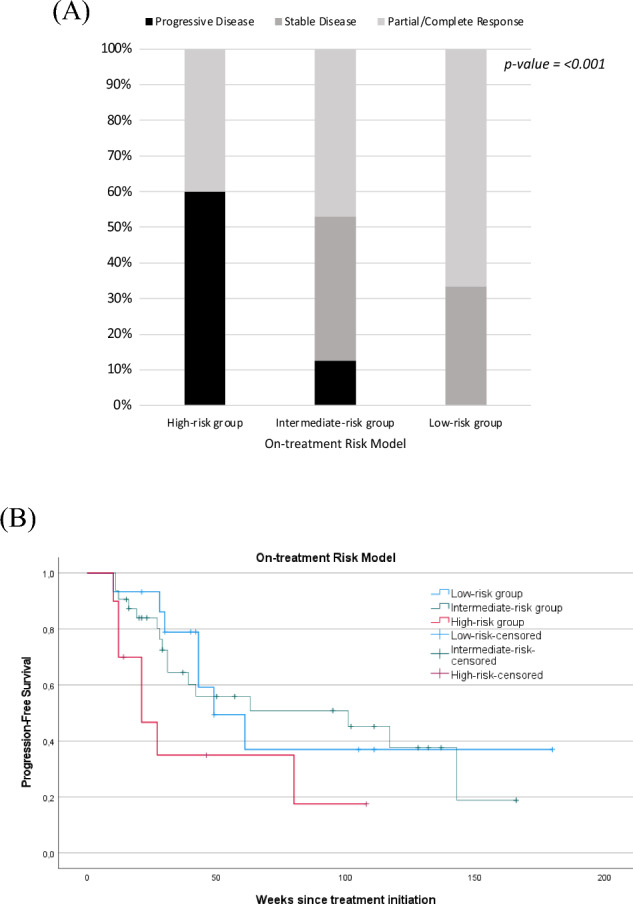
(**A**) Comparison of the primary treatment response and the risk groups of the on-treatment risk model. (**B**) Kaplan–Meier curve of the progression-free survival for the risk groups of the on-treatment model. The vertical axis represents the progression-free survival rate, and the horizontal axis represents the progression-free survival time (weeks) after treatment initiation

Based on the presence of above-specified factors, three risk groups were apparent: 15 patients were divided into the low-risk group by the presence of CRP flare-response/response and TRAE; 32 patients into the intermediate-risk group by the presence of only one factor; and 10 patients into the high-risk group by the absence of both factors. The primary treatment response was compared between the risk groups. At the initial radiological disease assessment, no progressive disease was observed in the low-risk group, while 60% (*n* = 6/10) of the high-risk group showed a primary disease progression (Fig. 4; *p* < 0.001). Two patients in the low-risk (*n* = 2/4) and intermediate risk (*n* = 2/4) groups had treatment discontinuation due to CTCAE Grade 3.

The median PFS amounted to 49 weeks (95% CI 24.9–73.0) for the favorable-risk group versus 21 weeks (95% CI 7.0–34.9) for the poor-risk group. The intermediate-risk group showed large differences in PFS with median by 101 weeks (95% CI 16.9–185.0 weeks) (*p* = 0.054).

## Discussion

In this study, we developed an on-treatment risk model for the primary treatment response for aRCC patients treated with immunotherapy combinations. This predictive model was constructed based on an activated immune system, reflected by on-treatment CRP kinetics and the early onset of TRAEs.

### Early CRP kinetics

Recent studies showed a strong association between on-treatment CRP kinetics and the treatment outcome in aRCC [9, 10, 12]. Fukuda et al. described the early changes of CRP levels from baseline to week 12 by aRCC patients treated with second-line immunotherapy and showed a significant association between CRP flare-response and improved survival outcomes [9]. Another published retrospective study by Klümper et al. found that the patients with emerging CRP flare kinetics had significantly longer median PFS [10, 11]. Recently, the previous definition of CRP flare-response by Fukuda et al. has been validated in a Phase III pivotal clinical trial for non-small cell lung cancer [12]. In our study, we investigated the predictive role of early CRP kinetics for the primary treatment response in aRCC patients treated with first-line immunotherapy combinations. We could validate the predictive association between CRP kinetics and the primary treatment response (*p* = 0.022). Regarding survival analysis, our study cohort showed no significant association with PFS and OS.

### Early onset of TRAEs

Early studies have begun understanding the molecular mechanism of immune-related adverse events (irAEs). It is suggested that irAEs occur as a bystander effect from activated T cells [17–19]. Recent studies showed a significant association between the occurrence of irAEs and improved treatment outcomes in several cancers, such as non-small lung cancer, melanoma, urothelial carcinoma, and renal cell carcinoma [20–22]. With increasing preference for IO-TKI combinations in the first-line treatment of renal cell carcinoma, the spectrum of adverse events also changed. Our study assessed the predictive value of the early onset of TRAEs for primary treatment response. We found that the patients with the early occurred TRAEs had a significantly lower risk for primary disease progression (*p* = 0.002), which aligns with previous studies.

On-treatment risk model for improved clinical monitoring in the era of IO-based combination treatment.

Although the CRP kinetics and occurrence of TRAEs have been evaluated as a clinical biomarker to predict the clinical outcome of immunotherapy in several studies, single biomarker measurements may not reflect the complex course of treatment monitoring and may have a limited predictive value. We hypothesized that the early CRP kinetics in combination with early onset of TRAEs may have higher predictive value for primary treatment response. For this purpose, we performed statistical assessments to construct an on-treatment risk model based on CRP kinetics and TRAEs. Importantly, we could demonstrate a significant risk model for the primary treatment response, which is illustrated in Fig. 4. An increased risk of the primary disease progression was observed by patients in the high-risk group without CRP flare-response/response and early onset of TRAEs (*p* < 0.001). There was no association between IMDC prognostic groups and the primary treatment response (Table 1). However, prospective studies are needed to evaluate the on-treatment changes of IMDC prognostic factors for the treatment response.

In summary, our findings support the consideration of the on-treatment CRP kinetics in combination with the early onset of TRAEs as a readily available tool to optimize the monitoring of patients and predict primary treatment outcome.

The limitations of our study are essentially impacted by the retrospective design and small sample size. Moreover, there was no identical time period for clinical visits and also for the first radiological assessment. Despite these limitations and limited available date, we provide a significant on-treatment risk model for the primary treatment response.

## Conclusion

Our analysis displays the predictive value of the above-mentioned on-treatment risk model based on CRP kinetics and the early onset of TRAEs, which can be easy to implement in clinical practice at no extra cost to optimize treatment monitoring in patients with advanced RCC. Further prospective studies are essential to confirm our findings and evaluate the changes of other immune-based factors. This will help to standardize the treatment monitoring for various types of cancer.

## Data Availability

Data are available upon reasonable request.
